# The cryptic diversity of hepadnavirus relatives

**DOI:** 10.1128/mbio.02541-25

**Published:** 2025-11-04

**Authors:** Zhen Gong, Guan-Zhu Han

**Affiliations:** 1 College of Life Sciences, Nanjing Normal University 224704, Nanjing, Jiangsu, China; National Institutes of Health, Bethesda, Maryland, USA

**Keywords:** hepadnavirus, virus diversity, virus evolution, phylogenetics

## Abstract

Enveloped hepadnaviruses, non-enveloped nackednaviruses, and the recently discovered non-enveloped proto-nackednaviruses are closely related reverse-transcribing DNA viruses. Yet, the host range and diversity of proto-nackednaviruses remain unclear. Here, we report the discovery of 31 proto-nackednaviruses (PnNVs) through deep genome-scale data mining. PnNVs are predominantly identified in distinct species of rotifers, implying the probable association between PnNVs and rotifers. Phylogenetic analyses show that PnNVs form at least five paraphyletic lineages sister to nackednaviruses and hepadnaviruses, unveiling the cryptic diversity of reverse-transcribing DNA viruses. This study provides insights into the origin, evolution, and diversity of reverse-transcribing DNA viruses.

**IMPORTANCE:**

Hepatitis B virus belongs to *Hepadnaviridae* and represents a serious threat to global public health. Recently, non-enveloped proto-nackednaviruses were discovered to be the closest relatives of *Hepadnaviridae*. However, the host range and diversity of proto-nackednaviruses remain unclear, impeding our understanding of the origin, evolution, and diversity of *Blubervirales* and reverse-transcribing DNA viruses in general. This work expands the hidden diversity of proto-nackednaviruses and indicates that rotifers might be their putative hosts. This work further traces the origin of *Blubervirales* back to 476 million years ago. These findings deepen our understanding of the evolution of reverse-transcribing DNA viruses.

## OBSERVATION

Based on the virus taxonomy of the International Committee on Taxonomy of Viruses, reverse-transcribing DNA viruses include two viral families, namely, *Hepadnaviridae* (in the order *Blubervirales*) and *Caulimoviridae* (in the order *Ortervirales*) (1–3). Hepadnaviruses and caulimoviruses are enveloped viruses that infect vertebrates and non-enveloped viruses that infect land plants, respectively (2–4). Hepadnaviruses and caulimoviruses are distantly related and appear to have originated independently (1).

Hepatitis B virus belongs to *Hepadnaviridae* and represents a serious threat to global public health. Hepadnaviruses possess small (3.0–3.3 kb), circular, partially double-stranded DNA genomes characterized by overlapping open reading frames (ORFs) encoding core (C), polymerase (P), surface (S), and X proteins. Their replication is initiated by the terminal protein (TP) of P and proceeded through reverse transcription of an RNA intermediate, pregenomic RNA (pgRNA), by reverse transcriptase (RT), and RNase H (RH) of P (5). Enveloped hepadnaviruses have been found in mammals, birds, reptiles, amphibians, and fish (6–12). Non-enveloped viruses closely related to hepadnaviruses have been reported recently, including nackednaviruses discovered in fish and proto-nackednaviruses identified in bat feces and permafrost (9, 13). Previous studies suggested an ancient origin for *Blubervirales* hundreds of millions of years ago (9, 13). Moreover, we discovered retroelements (known as HEART elements) closely related to *Blubervirales* in invertebrates, supporting the origin of hepadnaviruses from retroelements (14). Yet, the host range and diversity of proto-nackednaviruses remain mysterious, impeding our understanding of the origin, evolution, and diversity of *Blubervirales* and reverse-transcribing DNA viruses in general.

Here, we report the discovery of 31 proto-nackednaviruses (PnNVs) through deep mining of public genome-scale data. PnNVs are probably associated with rotifers. Phylogenetic analyses show PnNVs form several paraphyletic lineages sister to hepadnaviruses and nackednaviruses, expanding the diversity of *Blubervirales*. The origin of *Blubervirales* was traced back to 476 million years ago (MYA). Our findings have implications in understanding the origin, evolution, host range, and diversity of reverse-transcribing DNA viruses.

**Fig 1:**
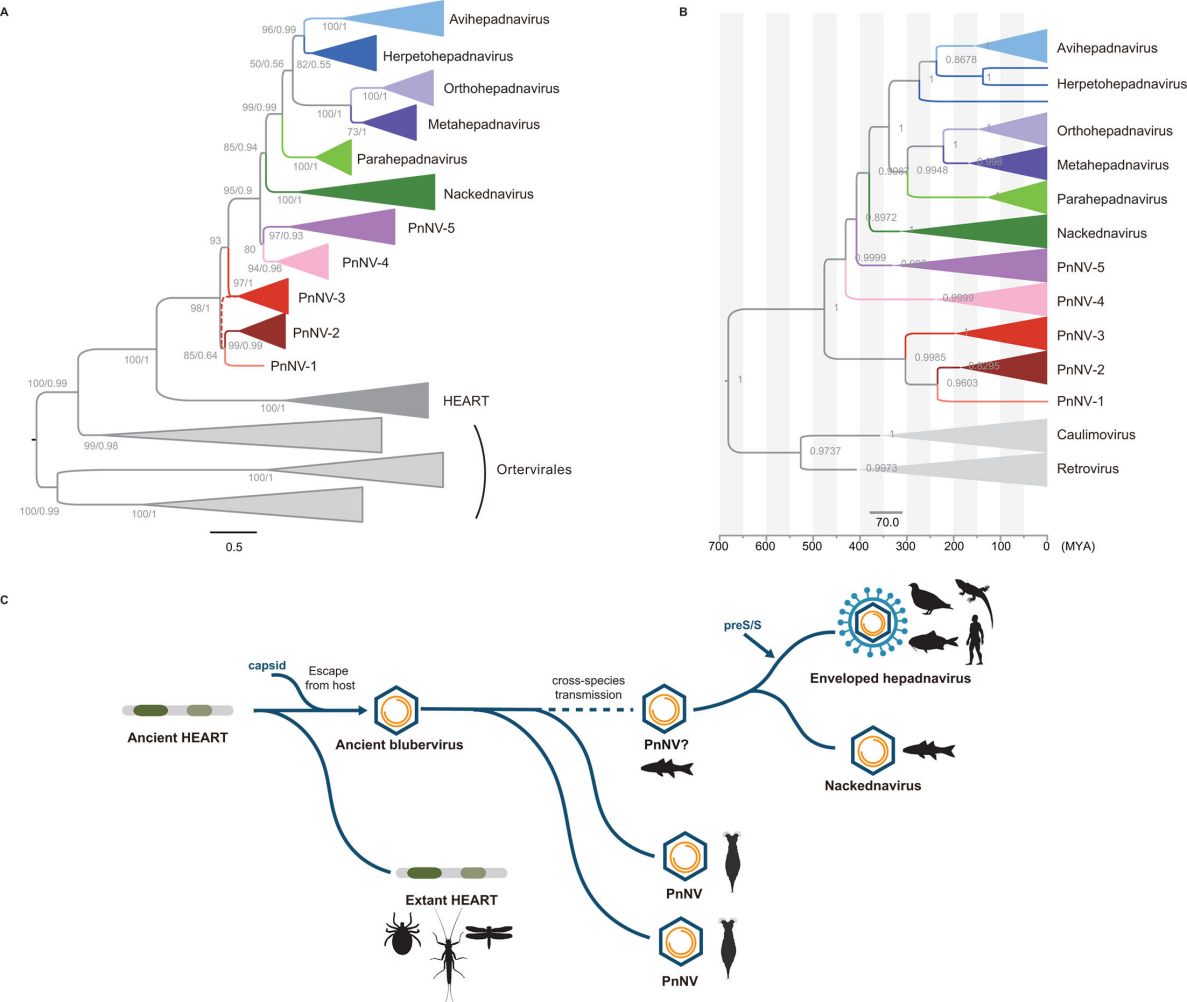
Origin and evolution of hepadnavirus-related elements. (**A**) Evolutionary relationship among hepadnavirus-related elements. The phylogenetic tree of HEART, proto-nackednaviruses, nackednaviruses, and hepadnaviruses was reconstructed based on RT proteins using a maximum likelihood method and a Bayesian method, with *Ortervirales* as outgroup. Ultrafast bootstrap supports/BPP values are shown near the nodes. The dashed branch indicates the topology inferred from the Bayesian method. (**B**) Time-calibrated Bayesian phylogeny of hepadnavirus-related elements. This maximum clade credibility tree was reconstructed based on P proteins using BEAST. BPP values are shown near the nodes. Scale bar indicates the time scale in MYA. (**C**) Evolutionary trajectory of hepadnaviruses. The dashed line indicates there is no solid evidence supporting the scenario of cross-species transmission of PnNVs.

Following our previous attempt to unveil the evolutionary history of hepadnaviruses (14), we performed deep mining of hepadnavirus-like elements in a wide range of genome-scale data, including 5,500 animal genomes, 4,912 metatranscriptomes, 12,053 global metagenomes, and 9,549 eukaryote transcriptome assemblies. We identified a total of 31 hepadnavirus-related elements in the genomes and transcriptomes of rotifers (*Rotaria macrura* and *Rotaria tardigrada*), the transcriptome of one amphipod (*Gammarus fossarum*), 1 freshwater metagenome, and 17 environmental metatranscriptomes (Table S1; Data Set S1). These elements can be clustered based on the nucleotide identity of 95% and the coverage of 85% into 23 virus operational taxonomic units (15). Phylogenetic analyses based on RT domains show that these hepadnavirus-like elements cluster with proto-nackednaviruses, nackednaviruses, and hepadnaviruses with strong support (ultrafast bootstrap [UFBoot] value = 98%, Bayesian posterior probability [BPP] = 1.00) (Fig. 1A; Fig. S1 to S3). These elements form five paraphyletic lineages with strong support (UFBoot value >90%, BPP >0.90), designated PnNV-1 to PnNV-5. Pliego virus and Toolik virus, the two recently discovered PnNVs (13), pertain to the PnNV-3 lineage. Moreover, PnNV lineages are sister to nackednaviruses and hepadnaviruses. Together, the discovery of PnNVs unveils the cryptic diversity of *Blubervirales*. These five PnNV lineages might be putatively classified as the family level.

**Fig 2:**
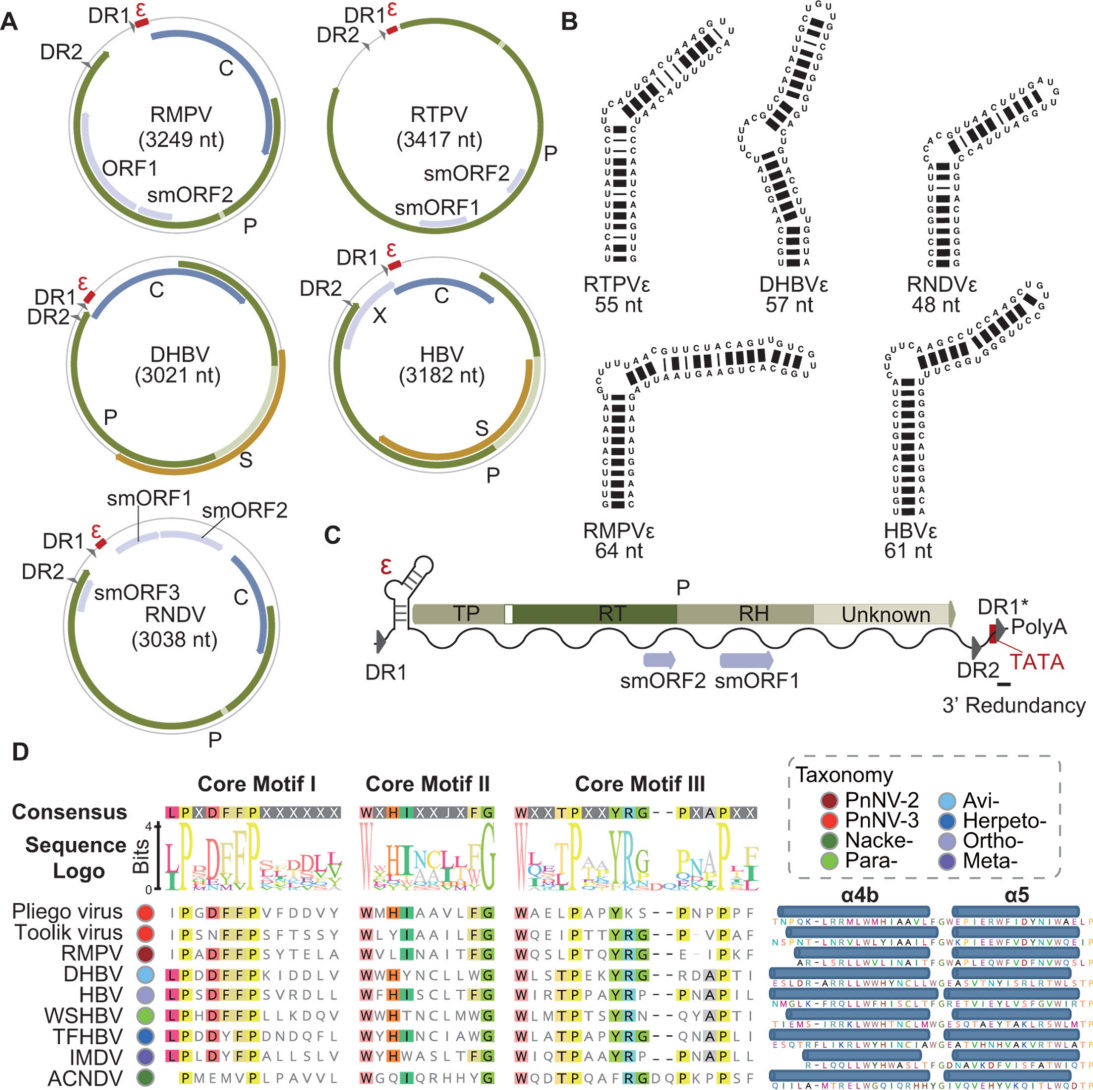
Comparative genome structure of proto-nackednaviruses (PnNVs), nackednaviruses, and hepadnaviruses. (**A**) Genome architectures of PnNVs (RMPV and RTPV), hepadnaviruses (DHBV and HBV), and a nackednavirus (RNDV). Light green stripes within P proteins indicate spacers. (**B**) Secondary structure of epsilons predicted by MC-Fold. (**C**) Genome organization of the linear pgRNA of RTPV. DR1^*^ indicates the second copy of DR1 resulted from reverse transcription. (**D**) Conservation in three core protein motifs (left) and predicted secondary structure α-helix 4b/5 (right) for nine representative viruses from PnNVs, nackednaviruses, and hepadnaviruses. The taxonomy of each virus is indicated by solid circles colored according to the color key at the top right corner. Abbreviations: Avi-, Avihepadnavirus; Herpeto-, Herpetohepadnavirus; Meta-, Metahepadnavirus; Nacke-, Nackednavirus; Ortho-, Orthohepadnavirus; Para-, Parahepadnavirus.

We assembled and annotated two complete genomes of PnNVs (Data Set S2) from rotifers: one putative pregenomic RNA (corresponding to ~3,249 nt in circular genome form) in the transcriptome of *R. tardigrada* (*R. tardigrada* PnNV [RTPV]) and one PnNV (~3417 nt) in the genome of *R. macrura* (*R. macrura* PnNV [RMPV]) (16). RTPV and RMPV pertain to PnNV-5 and PnNV-2 lineages, respectively (Fig. S2 and S3). The terminal redundancy and read mapping of the head-tail junction provide strong evidence for the circular and exogenous nature of RMPV (Fig. 2A). RMPV was predicted to encode four overlapping ORFs: two major ORFs encoding P and C proteins, ORF1 without detectable similarity to known proteins, and a small ORF (smORF2) of <300 nt. The RTPV genome comprises one major ORF encoding the P protein and two small ORFs (smORF1 and smORF2), but no C protein was predicted. Sequence-based and protein structure-based analyses did not identify high-confidence homologs to PnNV smORFs and RMPV ORF1. Like nackednaviruses and hepadnaviruses, the P proteins of RMPV and RTPV contain TP, RT, and RH domains. The spacer regions between TP and RT in PnNVs are much shorter than those in hepadnaviruses but similar to those in nackednaviruses (Fig. 2A). Two direct repeats (DR1 and DR2), which are essential for hepadnavirus replication, are present in both RMPV and RTPV (5). We also predicted RNA element epsilon (ε), which is crucial for reverse transcription priming, downstream of DR1 in both RMPV (64 nt) and RTPV (55 nt) (Fig. 2A) (17). Both RMPVε and RTPVε exhibit a hepadnaviral ε-like stem-bulge-stem-loop structure, indicating potential similarity in replication mechanisms among PnNVs, nackednaviruses, and hepadnaviruses (Fig. 2B) (17–19). For RTPV, we reconstructed a linear pgRNA sequence of 3,471 nt. This linear pgRNA contains other critical *cis*-regulatory elements, including TATA box and polyA tail (Fig. 2C). Notably, the *P* ORF of RTPV has a large extension of around 300 amino acids downstream of the RH domain, without known homologs (Fig. 2C). Production of pgRNA from the circular genome results in a short terminal redundancy of ~33 nt between TATA box and polyA tail, which contains a second copy of DR1 (DR1^*^). Furthermore, conserved motifs of C proteins were identified in PnNVs, and the predicted secondary structures also suggest conservation in α-helix 4b and α-helix 5 of PnNVs, hepadnaviruses, and nackednaviruses (Fig. 2D; Data Set S3) (20). However, no homolog to C protein was detected in RTPV, even when alternative genetic codes were used during ORF prediction. Read mapping analyses excluded the possibility of assembly mistakes (Fig. S5). Several possibilities might account for it: (i) RTPV lacks a conventional core protein; (ii) RTPV requires an undiscovered helper virus/segment; and (iii) it represents an mRNA rather than a pgRNA. Nevertheless, these results reveal the conservation and diversity in genome structures among *Blubervirales*.

Phylogenetic relationship and similarity in genome structures provide strong evidence that PnNVs are the closest relatives of hepadnaviruses and nackednaviruses. The discovery of complete PnNV pgRNA and genomes in different rotifer species indicates the probable association between PnNVs and rotifers, expanding the host range of *Blubervirales* (8–11).

Although rotifers acquire a substantial amount of genetic materials through horizontal gene transfer, these foreign genes are predominantly derived from bacteria, archaea, fungi, plants, and protists (21). Additionally, high abundance and sequencing average depth of RTPV and RMPV (the number of viral reads per million total filtered reads [RPM] >1) could largely reduce the possibility that PnNVs identified in rotifers were due to contamination during the sequencing process (Table 1). Nevertheless, further studies are still needed to verify the host range of PnNVs.

**TABLE 1:** Statistics of rotifer proto-nackednaviruses

Virus	Length (bp)	Min depth (*X*)	Max depth (*X*)	Average depth (*X*)	Read counts	Coverage (%)	Total filtered reads	RPM^*a*^
RMPV	3249	28	3,958	1,690	38,529	100	345,190,184	111.62
RTPV	3417	3	145	70	2,502	100	59,459,812	42.08

^*a*^ Viral abundance is represented by the number of viral reads per million total filtered reads (RPM).

To infer the evolutionary timescale of *Blubervirales*, we reconstructed a time-calibrated Bayesian phylogeny based on the P proteins using the integration time of an avihepadnavirus (eAHBV-FRY) in Neoaves genomes (69 MYA) as the calibration point (9, 11) (Fig. 1B; Fig. S4). The predicted divergence time between hepadnaviruses and nackednaviruses is 380 (95% HPD [highest posterior density]: 311–449) MYA, consistent with previous estimates (9), and PnNVs emerged at 476 (95% HPD: 381–565) MYA. The timescale suggests that *Blubervirales* likely infected vertebrates by cross-species transmission, as the inferred divergence time between PnNVs and hepadnaviruses/nackednaviruses (407 [95% HPD: 332–478] MYA) is much shorter than the divergence time between rotifers and vertebrates (about 686 MYA) (22) (Table S2).

Previously, we proposed a stepwise model for the origin and evolution of hepadnaviruses, evolving from retroelements to non-enveloped viruses to enveloped viruses (14). Building on the newly discovered diversity of proto-nackednaviruses, we proposed a refined stepwise evolutionary trajectory (Fig. 1C): PnNVs emerged from an escaped ancient invertebrate HEART retroelement. PnNVs infected vertebrates probably by cross-species transmission, leading to the origin of nackednaviruses in fish. Then, hepadnaviruses were derived from non-enveloped hepadnaviruses by gain of envelope. The discovery of PnNVs provides further insights into the origin and evolution of hepadnaviruses, bridging the evolutionary gap between retroelements and non-enveloped viruses.

PnNVs were identified in genome-scale data using similarity search and phylogenetic analysis combined approaches. Alignments were generated by MAFFT. Phylogenetic analyses were performed using FastTree, IQTREE, and MrBayes. Genome annotation involved sequence-based and structure-based searches by CD-Search, HHPred, and AlphaFold. Viral abundance is represented by the number of viral RPM. Reads were mapped to the viral genomes using Bowtie2. Time-calibrated Bayesian phylogeny was reconstructed by BEAST. See Supplemental Material for details.

## Data Availability

All data are included in the article and supplemental materials and have been deposited in figshare (10.6084/m9.figshare.29387867).

## SUPPLEMENTAL MATERIAL

Data set S1 mbio.02541-25-s0001.txt Nucleotide sequences of 32 PnNVs.

Data set S2 mbio.02541-25-s0002.txt Genome annotations of RTPV, RMPV, Toolik virus, and Pliego virus, related to Fig. 2.

Data set S3 mbio.02541-25-s0003.txt Alignment of core proteins from representative viruses, related to Fig. 2.

Data set S4 mbio.02541-25-s0004.txt Alignment of RT domains from representative retroelements, related to Fig. S1.

Data set S5 mbio.02541-25-s0005.txt Tree file of RT domains from representative retroelements, related to Fig. S1.

Data set S6 mbio.02541-25-s0006.txt Alignment of RT domains from hepadnavirus-related elements, related to Fig. 1A.

Data set S7 mbio.02541-25-s0007.txt Maximum-likelihood tree file of RT domains from hepadnavirus-related elements, related to Fig. 1A.

Data set S8 mbio.02541-25-s0008.txt Bayesian tree file of RT domains from hepadnavirus-related elements, related to Fig. 1. 1A.

Data set S9 mbio.02541-25-s0009.txt Alignment and time-calibrated tree file of P proteins from hepadnavirus-related elements, related to Fig. 1B.

Supplemental material mbio.02541-25-s0010.pdf Supplemental methods, Fig. S1-S5, and Tables S1 and S2.

ASM does not own the copyrights to Supplemental Material that may be linked to, or accessed through, an article. The authors have granted ASM a non-exclusive, world-wide license to publish the Supplemental Material files. Please contact the corresponding author directly for reuse.
