# Supplementary material for: The cryptic diversity of hepadnavirus relatives
Source: mBio. 2025 Nov 4;16(12):e02541-25. doi: 10.1128/mbio.02541-25 (PMC12691608; doi:10.1128/mbio.02541-25)
Supplement: Supplemental material — Supplemental methods, Fig. S1-S5, and Tables S1 and S2. [file mbio.02541-25-s0010.pdf]

Supplementary Materials

The cryptic diversity of hepadnavirus relatives

Zhen Gong[1,*] and Guan-Zhu Han[1,*]

[1]College of Life Sciences, Nanjing Normal University, Nanjing, Jiangsu 210023, China

[*] To whom correspondence should be addressed. E-mail: guanzhu@njnu.edu.cn (G.-Z.H.) or gongzhen@nnu.edu.cn (Z.G.)

Supplementary Methods

Identification of PnNVs

We conducted similarity search and phylogenetic analysis combined approach to identify hepadnavirus-like elements within 5500 animal genomes from NCBI, 4912 meta-transcriptomes from JGI, 12,053 global metagenomes from JGI, and 9549 eukaryote transcriptome assemblies from NCBI. First, we used tBLASTn and PSI-BLAST algorithms to search against genomes and transcriptomes, with RT proteins of representative hepadnaviruses as queries and an *e* cut-off value of 10^{-5} [1-2]. Next, significant hits were retrieved and subjected to large-scale phylogenetic analyses with representative retroelements using an approximate maximum likelihood method implemented in FastTree [3-4]. Sequences that clustered with hepadnaviruses were extracted for the next round of phylogenetic analyses. We identified a total of 31 proto-nackednaviruses (PnNVs) within rotifer genomes and transcriptomes, amphipod transcriptomes, one global metagenome, and 17 environmental meta-transcriptomes (Supplementary Table S1 and Dataset S1). These 31 PnNVs were further grouped into 23 virus operational taxonomic units (vOTUs) by CD-HIT based on a clustering criterion roughly corresponding to the species level: >95% genome-wide average nucleotide identity and >85% coverage [5-6].

Genome annotation of PnNVs

ORFs of PnNVs with >3,000 nt were predicted by Geneious, with a length cut-off of 150 nt [7]. Domains were annotated using CD-Search and InterproScan [8-9]. Core proteins were predicted by Phyre2 and HHpred [10-11]. Protein 3D structure-based searches were performed using AlphaFold, Dali, and Foldseek [12-14]. Due to low conservation in RNA element epsilon (ε), we were unable to identify ε of PnNVs through similarity search or sequence alignment. Thus, MC-Fold was used for 5' terminal region of PnNVs to predict potential ε elements with a hepadnaviral ε like stem-bulge-stem-loop structure, with window size ranging from 50 to 80 nt [15]. Protein secondary structure of core proteins was predicted with Jpred [16]. The alignment of

core proteins from representative viruses was generated by the L-INS-I method in MAFFT and improved by the iterative refinement method with a maxiterate of 1,000 (Supplementary Dataset S3) [17]. Visualization of genome structures and alignments was performed by Geneious [7].

Phylogenetic analyses of PnNVs

To explore evolutionary relationships among PnNVs, nackednaviruses, and hepadnaviruses, we performed phylogenetic analyses based on RT proteins. The alignment was generated by the L-INS-I method in MAFFT, followed by manual refinement to remove ambiguous sites [17]. The length of the final alignment is 284. Phylogenetic analysis was performed using a maximum likelihood method implemented in IQ-TREE [18]. The best-fit amino acid substitution model was detected using the ModelFinder algorithm [19]. UFBoot supports for nodes were assessed using the ultrafast bootstrap (UFBoot) approach with 1,000 replicates [20]. To provide further support for the phylogeny, we also reconstructed a Bayesian phylogenetic tree based on the same RT alignment using MrBayes [21]. The phylogeny was rooted by using elements from Ortervirales as outgroups.

Time-calibration of the virus phylogeny

To infer the divergence time of hepadnavirus relatives, we reconstructed a time-calibrated Bayesian phylogeny based on P proteins. P protein sequences of PnNVs identified in this study were added to the alignment used in the previous study using MAFFT, followed by manual refinement to remove ambiguous sites [17,22]. The age of eAHBV-FRY elements (mean of 69 MYA and standard deviation of 1) was used as the calibration point [23]. The phylogeny was reconstructed using BEAST v1.10.4 with the amino acid substitution model selected by ProtTest (JTT+G), a calibrated Yule speciation model, and the uncorrelated relaxed clock with log-normal distribution [24-25]. A single chain was run for 80 million generations, with parameters sampled every 8,000 steps. Following convergence assessment with Tracer [26], the maximum clade

credibility (MCC) tree was summarized using TreeAnnotator, with a burn-in of 20 million states (25% of the total chain length).

Virus abundance analyses

The quality of raw reads of *R. tardigrada* transcriptome and *R. macrura* genome were first controlled using Trimmomatic [27], and rRNA reads in the transcriptome library were removed by mapping to the rRNA reference database from SILVA (https://www.arb-silva.de/) using Bowtie2 [28]. Filtered reads were mapped to the viral genomes of RTPV and RMPV using Bowtie2 (options '--end-to-end' and '-sensitive'). Virus abundance was quantified as the number of viral mapped reads per million total filtered reads (RPM) in the library.

Supplementary Figures

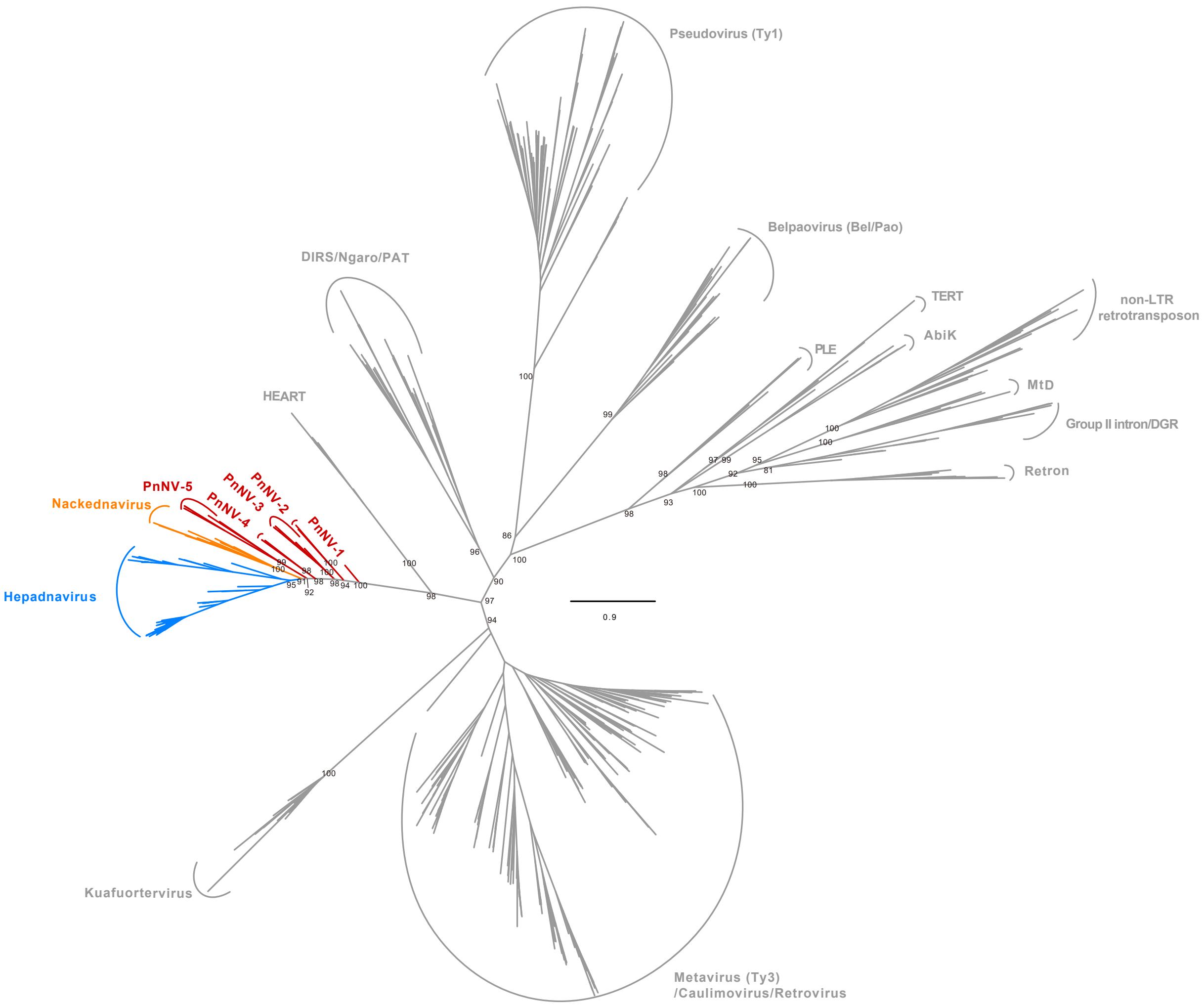

Supplementary Figure S1. Phylogenetic relationship among representative retroelements. The unrooted phylogenetic relationship was reconstructed based on RT proteins using a maximum likelihood method. UFBoot support values are shown near the nodes.

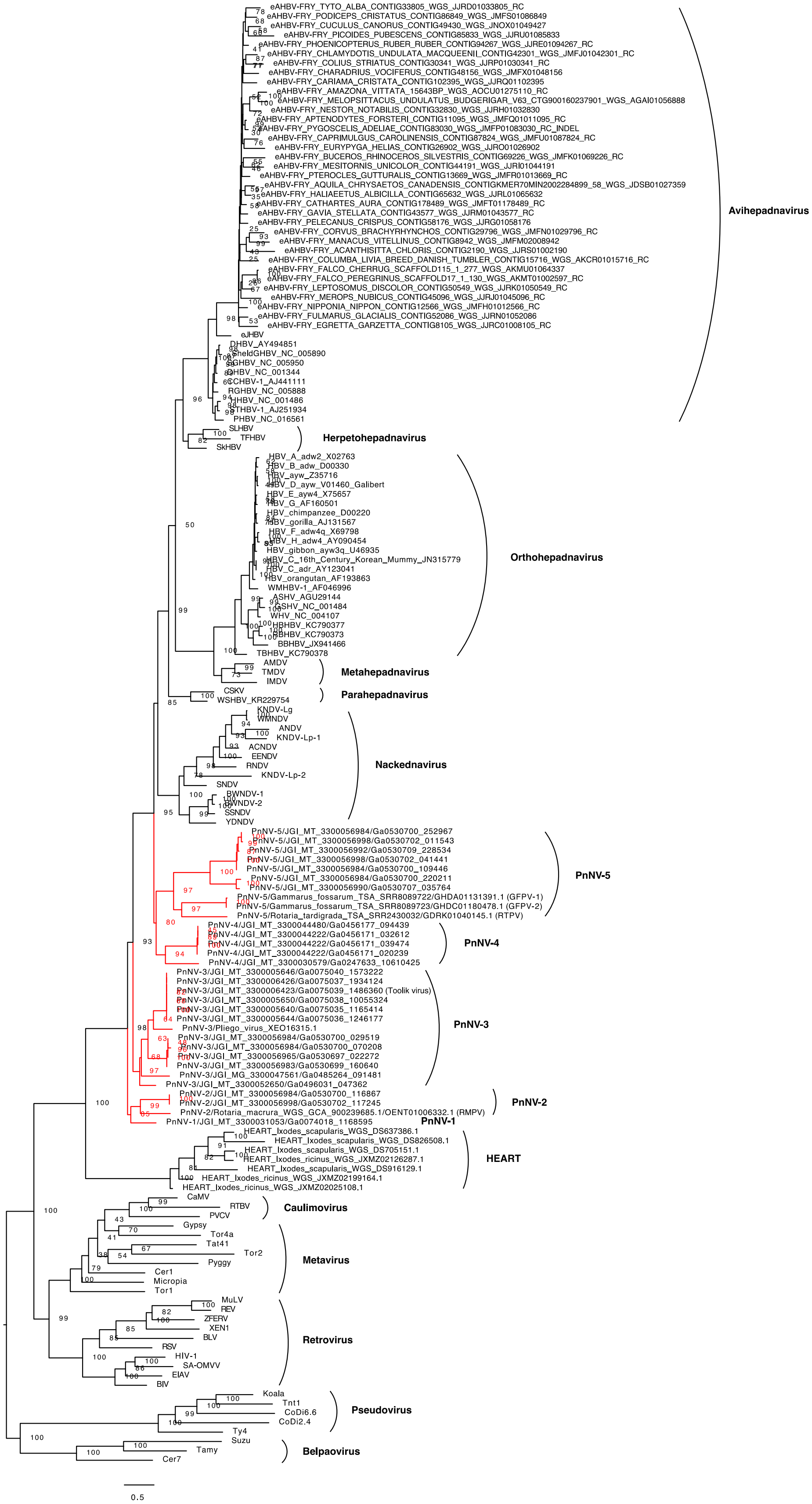

eAHBV-FRY_TYTO_ALBA_CONTIG33805_WGS_JJRD01033805_RC
eAHBV-FRY_PODICEPS_CRISTATUS_CONTIG86849_WGS_JMFS01086849
eAHBV-FRY_CUCULUS_CANORUS_CONTIG49430_WGS_JNOX01049427
eAHBV-FRY_PICOIDES_PUBESCENS_CONTIG85833_WGS_JJRU01085833
eAHBV-FRY_PHOENICOPTERUS_RUBER_RUBER_CONTIG94267_WGS_JJRE01094267_RC
eAHBV-FRY_CHLAMYDOTIS_UNDULATA_MACQUEENII_CONTIG42301_WGS_JMFJ01042301_RC
eAHBV-FRY_COLIUS_STRIATUS_CONTIG30341_WGS_JJRP01030341_RC
eAHBV-FRY_CHARADRIUS_VOCIFERUS_CONTIG48156_WGS_JMFX01048156
eAHBV-FRY_CARIAMA_CRISTATA_CONTIG102395_WGS_JJRQ01102395
eAHBV-FRY_AMAZONA_VITTATA_15643BP_WGS_AOCU01275110_RC
eAHBV-FRY_MELOPSITTACUS_UNDULATUS_BUDGERIGAR_V63_CTG900160237901_WGS_AGAI01056888
eAHBV-FRY_NESTOR_NOTABILIS_CONTIG32830_WGS_JJRH01032830
eAHBV-FRY_APTENODYTES_FORSTERI_CONTIG11095_WGS_JMFQ01011095_RC
eAHBV-FRY_PYGOSCELIS_ADELIAE_CONTIG83030_WGS_JMFP01083030_RC_INDEL
eAHBV-FRY_CAPRIMULGUS_CAROLINENSIS_CONTIG87824_WGS_JMFU01087824_RC
eAHBV-FRY_EURYPYGA_HELIAS_CONTIG26902_WGS_JJRO01026902
eAHBV-FRY_BUCEROS_RHINOCEROS_SILVESTRIS_CONTIG69226_WGS_JMFK01069226_RC
eAHBV-FRY_MESITORNIS_UNICOLOR_CONTIG44191_WGS_JJRI01044191
eAHBV-FRY_PTEROCLES_GUTTURALIS_CONTIG13669_WGS_JMFR01013669_RC
eAHBV-FRY_AQUILA_CHRYSAETOS_CANADENSIS_CONTIGKMER70MIN2002284899_58_WGS_JDSB01027359
eAHBV-FRY_HALIAEETUS_ALBICILLA_CONTIG65632_WGS_JJRL01065632
eAHBV-FRY_CATHARTES_AURA_CONTIG178489_WGS_JMFT01178489_RC
eAHBV-FRY_GAVIA_STELLATA_CONTIG43577_WGS_JJRM01043577_RC
eAHBV-FRY_PELECANUS_CRISPUS_CONTIG58176_WGS_JJRG01058176
eAHBV-FRY_CORVUS_BRACHYRHYNCHOS_CONTIG29796_WGS_JMFN01029796_RC
eAHBV-FRY_MANACUS_VITELLINUS_CONTIG8942_WGS_JMFM02008942
eAHBV-FRY_ACANTHISITTA_CHLORIS_CONTIG2190_WGS_JJRS01002190
eAHBV-FRY_COLUMBA_LIVIA_BREED_DANISH_TUMBLER_CONTIG15716_WGS_AKCR01015716_RC
eAHBV-FRY_FALCO_CHERRUG_SCAFFOLD115_1_277_WGS_AKMU01064337
eAHBV-FRY_FALCO_PEREGRINUS_SCAFFOLD17_1_130_WGS_AKMT01002597_RC
eAHBV-FRY_LEPTOSOMUS_DISCOLOR_CONTIG50549_WGS_JJRK01050549_RC
eAHBV-FRY_MEROPS_NUBICUS_CONTIG45096_WGS_JJRJ01045096_RC
eAHBV-FRY_NIPPONIA_NIPPON_CONTIG12566_WGS_JMFH01012566_RC
eAHBV-FRY_FULMARUS_GLACIALIS_CONTIG52086_WGS_JJRN01052086
eAHBV-FRY_EGRETTA_GARZETTA_CONTIG8105_WGS_JJRC01008105_RC
eJHBV
DHBV_AY494851
SheldGHBV_NC_005890
SGHBV_NC_005950
DHBV_NC_001344
CCHBV-1_AJ441111
RGHBV_NC_005888
HHBV_NC_001486
STHBV-1_AJ251934
PHBV_NC_016561
Avihepadnavirus
SLHBV
TFHBV
SkHBV
Herpetohepadnavirus
HBV_A_adw2_X02763
HBV_B_adw_D00330
HBV_ayw_Z35716
HBV_D_ayw_V01460_Galibert
HBV_E_ayw4_X75657
HBV_G_AF160501
HBV_chimpanzee_D00220
HBV_gorilla_AJ131567
HBV_F_adw4q_X69798
HBV_H_adw4_AY090454
HBV_gibbon_ayw3q_U46935
HBV_C_16th_Century_Korean_Mummy_JN315779
HBV_C_adr_AY123041
HBV_orangutan_AF193863
WMHBV-1_AF046996
ASHV_AGU29144
GSHV_NC_001484
WHV_NC_004107
HBHBV_KC790377
RBHBV_KC790373
BBHBV_JX941466
TBHBV_KC790378
Orthohepadnavirus
AMDV
TMDV
IMDV
Metahepadnavirus
CSKV
WSHBV_KR229754
Parahepadnavirus
KNDV-Lg
WMNDV
ANDV
KNDV-Lp-1
ACNDV
EENDV
RNDV
KNDV-Lp-2
SNDV
BWNDV-1
BWNDV-2
SSNDV
YDNDV
Nackednavirus
PnNV-5/JGI_MT_3300056984/Ga0530700_252967
PnNV-5/JGI_MT_3300056998/Ga0530702_011543
PnNV-5/JGI_MT_3300056992/Ga0530709_228534
PnNV-5/JGI_MT_3300056998/Ga0530702_041441
PnNV-5/JGI_MT_3300056984/Ga0530700_109446
PnNV-5/JGI_MT_3300056984/Ga0530700_220211
PnNV-5/JGI_MT_3300056990/Ga0530707_035764
PnNV-5/Gammarus_fossarum_TSA_SRR8089722/GHDA01131391.1 (GFPV-1)
PnNV-5/Gammarus_fossarum_TSA_SRR8089723/GHDC01180478.1 (GFPV-2)
PnNV-5/Rotaria_tardigrada_TSA_SRR2430032/GDRK01040145.1 (RTPV)
PnNV-5
PnNV-4/JGI_MT_3300044480/Ga0456177_094439
PnNV-4/JGI_MT_3300044222/Ga0456171_032612
PnNV-4/JGI_MT_3300044222/Ga0456171_039474
PnNV-4/JGI_MT_3300044222/Ga0456171_020239
PnNV-4/JGI_MT_3300030579/Ga0247633_10610425
PnNV-4
PnNV-3/JGI_MT_3300005646/Ga0075040_1573222
PnNV-3/JGI_MT_3300006426/Ga0075037_1934124
PnNV-3/JGI_MT_3300006423/Ga0075039_1486360 (Toolik virus)
PnNV-3/JGI_MT_3300005650/Ga0075038_10055324
PnNV-3/JGI_MT_3300005640/Ga0075035_1165414
PnNV-3/JGI_MT_3300005644/Ga0075036_1246177
PnNV-3/Pliego_virus_XEO16315.1
PnNV-3/JGI_MT_3300056984/Ga0530700_029519
PnNV-3/JGI_MT_3300056984/Ga0530700_070208
PnNV-3/JGI_MT_3300056965/Ga0530697_022272
PnNV-3/JGI_MT_3300056983/Ga0530699_160640
PnNV-3/JGI_MG_3300047561/Ga0485264_091481
PnNV-3/JGI_MT_3300052650/Ga0496031_047362
PnNV-3
PnNV-2/JGI_MT_3300056984/Ga0530700_116867
PnNV-2/JGI_MT_3300056998/Ga0530702_117245
PnNV-2/Rotaria_macrura_WGS_GCA_900239685.1/OENT01006332.1 (RMPV)
PnNV-2
PnNV-1/JGI_MT_3300031053/Ga0074018_1168595
PnNV-1
HEART_Ixodes_scapularis_WGS_DS637386.1
HEART_Ixodes_scapularis_WGS_DS826508.1
HEART_Ixodes_scapularis_WGS_DS705151.1
HEART_Ixodes_ricinus_WGS_JXMZ02126287.1
HEART_Ixodes_scapularis_WGS_DS916129.1
HEART_Ixodes_ricinus_WGS_JXMZ02199164.1
HEART_Ixodes_ricinus_WGS_JXMZ02025108.1
HEART
CaMV
RTBV
PVCV
Caulimovirus
Gypsy
Tor4a
Tat41
Tor2
Pyggy
Cer1
Micropia
Tor1
Metavirus
MuLV
REV
ZFERV
XEN1
BLV
RSV
HIV-1
SA-OMVV
EIAV
BIV
Retrovirus
Koala
Tnt1
CoDi6.6
CoDi2.4
Ty4
Pseudovirus
Suzu
Tamy
Cer7
Belpaovirus
0.5

Supplementary Figure S2. Maximum likelihood phylogenetic relationship among hepadnavirus-related elements. The phylogenetic tree of HEART, proto-nackednaviruses, nackednaviruses, and hepadnaviruses was reconstructed based on RT proteins using a maximum likelihood method, with Ortervirales as outgroups. This is the enlarged version of Figure 1A. UFBoot supports are shown near the nodes. Five PnNV lineages are highlighted by red branches.

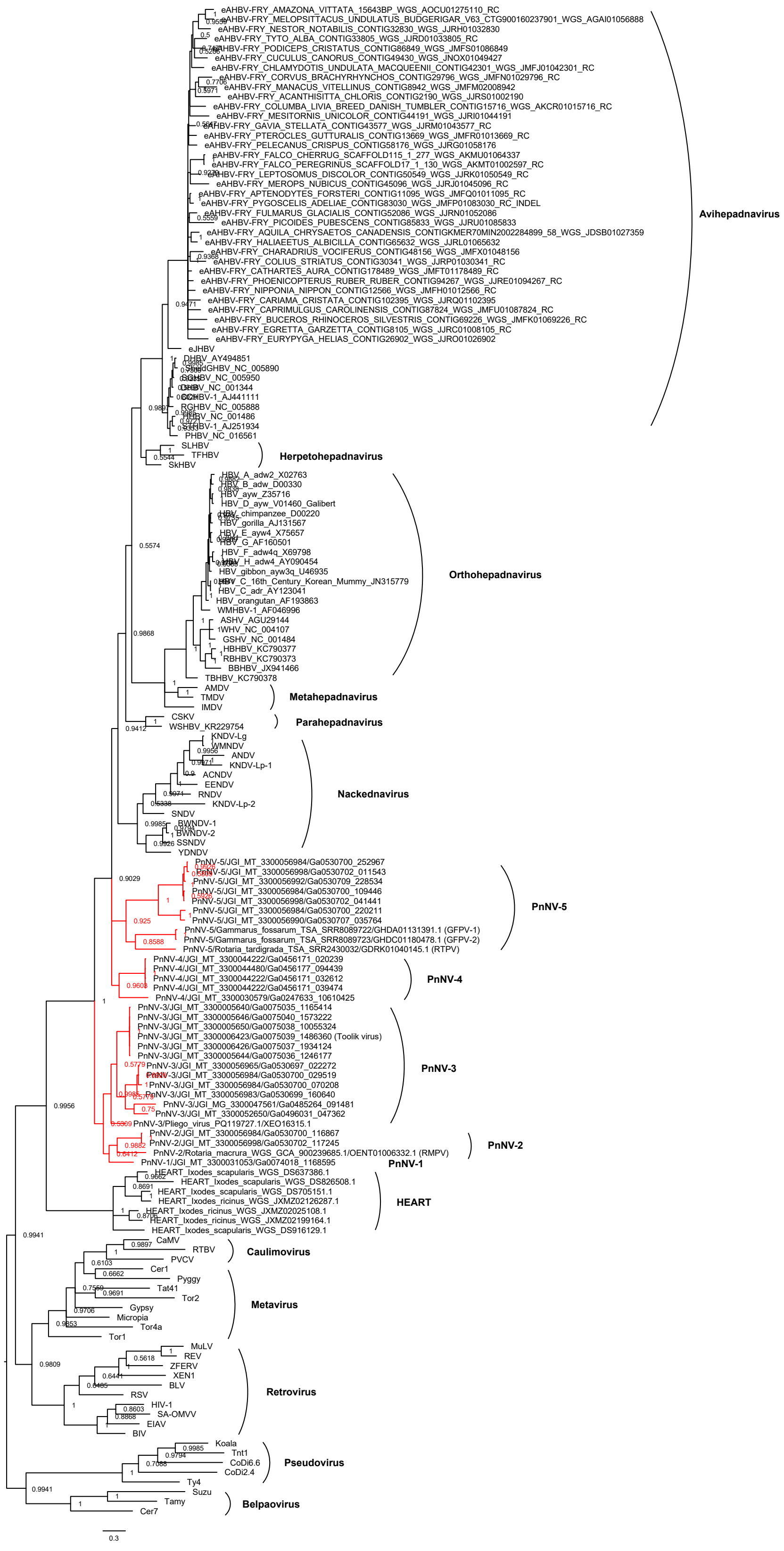

eAHBV-FRY_AMAZONA_VITTATA_15643BP_WGS_AOCU01275110_RC
eAHBV-FRY_MELOPSITTACUS_UNDULATUS_BUDGERIGAR_V63_CTG900160237901_WGS_AGAI01056888
eAHBV-FRY_NESTOR_NOTABILIS_CONTIG32830_WGS_JJRH01032830
eAHBV-FRY_TYTO_ALBA_CONTIG33805_WGS_JJRD01033805_RC
eAHBV-FRY_PODICEPS_CRISTATUS_CONTIG86849_WGS_JMFS01086849
eAHBV-FRY_CUCULUS_CANORUS_CONTIG49430_WGS_JNOX01049427
eAHBV-FRY_CHLAMYDOTIS_UNDULATA_MACQUEENII_CONTIG42301_WGS_JMFJ01042301_RC
eAHBV-FRY_CORVUS_BRACHYRHYNCHOS_CONTIG29796_WGS_JMFN01029796_RC
eAHBV-FRY_MANACUS_VITELLINUS_CONTIG8942_WGS_JJRM02008942
eAHBV-FRY_ACANTHISITTA_CHLORIS_CONTIG2190_WGS_JJRS01002190
eAHBV-FRY_COLUMBA_LIVIA_BREED_DANISH_TUMBLER_CONTIG15716_WGS_AKCR01015716_RC
eAHBV-FRY_MESITORNIS_UNICOLOR_CONTIG44191_WGS_JJRI01044191
eAHBV-FRY_GAVIA_STELLATA_CONTIG43577_WGS_JJRM01043577_RC
eAHBV-FRY_PTEROCLES_GUTTURALIS_CONTIG13669_WGS_JMFR01013669_RC
eAHBV-FRY_PELECANUS_CRISPUS_CONTIG58176_WGS_JJRG01058176
eAHBV-FRY_FALCO_CHERRUG_SCAFFOLD115_1_277_WGS_AKMU01064337
eAHBV-FRY_FALCO_PEREGRINUS_SCAFFOLD17_1_130_WGS_AKMT01002597_RC
eAHBV-FRY_LEPTOSOMUS_DISCOLOR_CONTIG50549_WGS_JJRK01050549_RC
eAHBV-FRY_MEROPS_NUBICUS_CONTIG45096_WGS_JJRJ01045096_RC
eAHBV-FRY_APTENODYTES_FORSTERI_CONTIG11095_WGS_JMFQ01011095_RC
eAHBV-FRY_PYGOSCELIS_ADELIAE_CONTIG83030_WGS_JMFP01083030_RC_INDEL
eAHBV-FRY_FULMARUS_GLACIALIS_CONTIG52086_WGS_JJRN01052086
eAHBV-FRY_PICOIDES_PUBESCENS_CONTIG85833_WGS_JJRU01085833
eAHBV-FRY_AQUILA_CHRYSAETOS_CANADENSIS_CONTIGKMER70MIN2002284899_58_WGS_JDSB01027359
eAHBV-FRY_HALIAEETUS_ALBICILLA_CONTIG65632_WGS_JJRL01065632
eAHBV-FRY_CHARADRIUS_VOCIFERUS_CONTIG48156_WGS_JMFX01048156
eAHBV-FRY_COLIUS_STRIATUS_CONTIG30341_WGS_JJRP01030341_RC
eAHBV-FRY_CATHARTES_AURA_CONTIG178489_WGS_JMFT01178489_RC
eAHBV-FRY_PHOENICOPTERUS_RUBER_RUBER_CONTIG94267_WGS_JJRE01094267_RC
eAHBV-FRY_NIPPONIA_NIPPON_CONTIG12566_WGS_JMFH01012566_RC
eAHBV-FRY_CARIAMA_CRISTATA_CONTIG102395_WGS_JJRQ01102395
eAHBV-FRY_CAPRIMULGUS_CAROLINENSIS_CONTIG87824_WGS_JMFU01087824_RC
eAHBV-FRY_BUCEROS_RHINOCEROS_SILVESTRIS_CONTIG69226_WGS_JMFK01069226_RC
eAHBV-FRY_EGRETTA_GARZETTA_CONTIG8105_WGS_JJRC01008105_RC
eAHBV-FRY_EURYPYGA_HELIAS_CONTIG26902_WGS_JJRO01026902
eJHBV
DHBV_AY494851
RGHBV_NC_005888
HHBV_NC_001486
PHBV_NC_016561
Avihepadnavirus
SLHBV
TFHBV
SkHBV
Herpetohepadnavirus
HBV_A_adw2_X02763
HBV_B_adw_D00330
HBV_ayw_Z35716
HBV_D_ayw_V01460_Galibert
HBV_chimpanzee_D00220
HBV_gorilla_AJ131567
HBV_E_ayw4_X75657
HBV_G_AF160501
HBV_F_adw4q_X69798
HBV_H_adw4_AY090454
HBV_gibbon_ayw3q_U46935
HBV_C_16th_Century_Korean_Mummy_JN315779
HBV_C_adr_AY123041
HBV_orangutan_AF193863
WMHBV-1_AF046996
ASHV_AGU29144
WHV_NC_004107
GSHV_NC_001484
HBHBV_KC790377
RBHBV_KC790373
BBHBV_JX941466
TBHBV_KC790378
Orthohepadnavirus
AMDV
TMDV
IMDV
Metahepadnavirus
CSKV
WSHBV_KR229754
Parahepadnavirus
KNDV-Lg
WMNDV
ANDV
KNDV-Lp-1
ACNDV
EENDV
RNDV
KNDV-Lp-2
SNDV
BWNDV-1
BWNDV-2
SSNDV
YDNDV
Nackednavirus
PnNV-5/JGI_MT_3300056984/Ga0530700_252967
PnNV-5/JGI_MT_3300056998/Ga0530702_011543
PnNV-5/JGI_MT_3300056992/Ga0530709_228534
PnNV-5/JGI_MT_3300056984/Ga0530700_109446
PnNV-5/JGI_MT_3300056998/Ga0530702_041441
PnNV-5/JGI_MT_3300056984/Ga0530700_220211
PnNV-5/JGI_MT_3300056990/Ga0530707_035764
PnNV-5/Gammarus_fossarum_TSA_SRR8089722/GHDA01131391.1 (GFPV-1)
PnNV-5/Gammarus_fossarum_TSA_SRR8089723/GHDC01180478.1 (GFPV-2)
PnNV-5/Rotaria_tardigrada_TSA_SRR2430032/GDRK01040145.1 (RTPV)
PnNV-5
PnNV-4/JGI_MT_3300044222/Ga0456171_020239
PnNV-4/JGI_MT_3300044480/Ga0456177_094439
PnNV-4/JGI_MT_3300044222/Ga0456171_032612
PnNV-4/JGI_MT_3300044222/Ga0456171_039474
PnNV-4/JGI_MT_3300030579/Ga0247633_10610425
PnNV-4
PnNV-3/JGI_MT_3300005640/Ga0075035_1165414
PnNV-3/JGI_MT_3300005646/Ga0075040_1573222
PnNV-3/JGI_MT_3300005650/Ga0075038_10055324
PnNV-3/JGI_MT_3300006423/Ga0075039_1486360 (Toolik virus)
PnNV-3/JGI_MT_3300006426/Ga0075037_1934124
PnNV-3/JGI_MT_3300005644/Ga0075036_1246177
PnNV-3/JGI_MT_3300056965/Ga0530697_022272
PnNV-3/JGI_MT_3300056984/Ga0530700_029519
PnNV-3/JGI_MT_3300056984/Ga0530700_070208
PnNV-3/JGI_MT_3300056983/Ga0530699_160640
PnNV-3/JGI_MG_3300047561/Ga0485264_091481
PnNV-3/JGI_MT_3300052650/Ga0496031_047362
PnNV-3/Pliego_virus_PQ119727.1/XEO16315.1
PnNV-3
PnNV-2/JGI_MT_3300056984/Ga0530700_116867
PnNV-2/JGI_MT_3300056998/Ga0530702_117245
PnNV-2/Rotaria_macrura_WGS_GCA_900239685.1/OENT01006332.1 (RMPV)
PnNV-2
PnNV-1/JGI_MT_3300031053/Ga0074018_1168595
PnNV-1
HEART_Ixodes_scapularis_WGS_DS637386.1
HEART_Ixodes_scapularis_WGS_DS826508.1
HEART_Ixodes_scapularis_WGS_DS705151.1
HEART_Ixodes_ricinus_WGS_JXMZ02126287.1
HEART_Ixodes_ricinus_WGS_JXMZ02025108.1
HEART_Ixodes_ricinus_WGS_JXMZ02199164.1
HEART_Ixodes_scapularis_WGS_DS916129.1
HEART
CaMV
RTBV
Caulimovirus
PVCV
Cer1
Pyggy
Tat41
Tor2
Gypsy
Micropia
Tor4a
Tor1
Metavirus
MuLV
REV
ZFERV
XEN1
BLV
RSV
HIV-1
SA-OMVV
EIAV
BIV
Retrovirus
Koala
Tnt1
CoDi6.6
CoDi2.4
Ty4
Pseudovirus
Suzu
Tamy
Cer7
Belpaovirus
0.3

Supplementary Figure S3. Bayesian phylogenetic relationship among hepadnavirus-related elements. The phylogenetic tree of HEART, proto-nackednaviruses, nackednaviruses, and hepadnaviruses was reconstructed based on RT proteins using a Bayesian method, with Ortervirales as outgroups. This is the enlarged version of Figure 1A. Bayesian posterior probability values are shown near the nodes. Five PnNV lineages are highlighted by red branches.

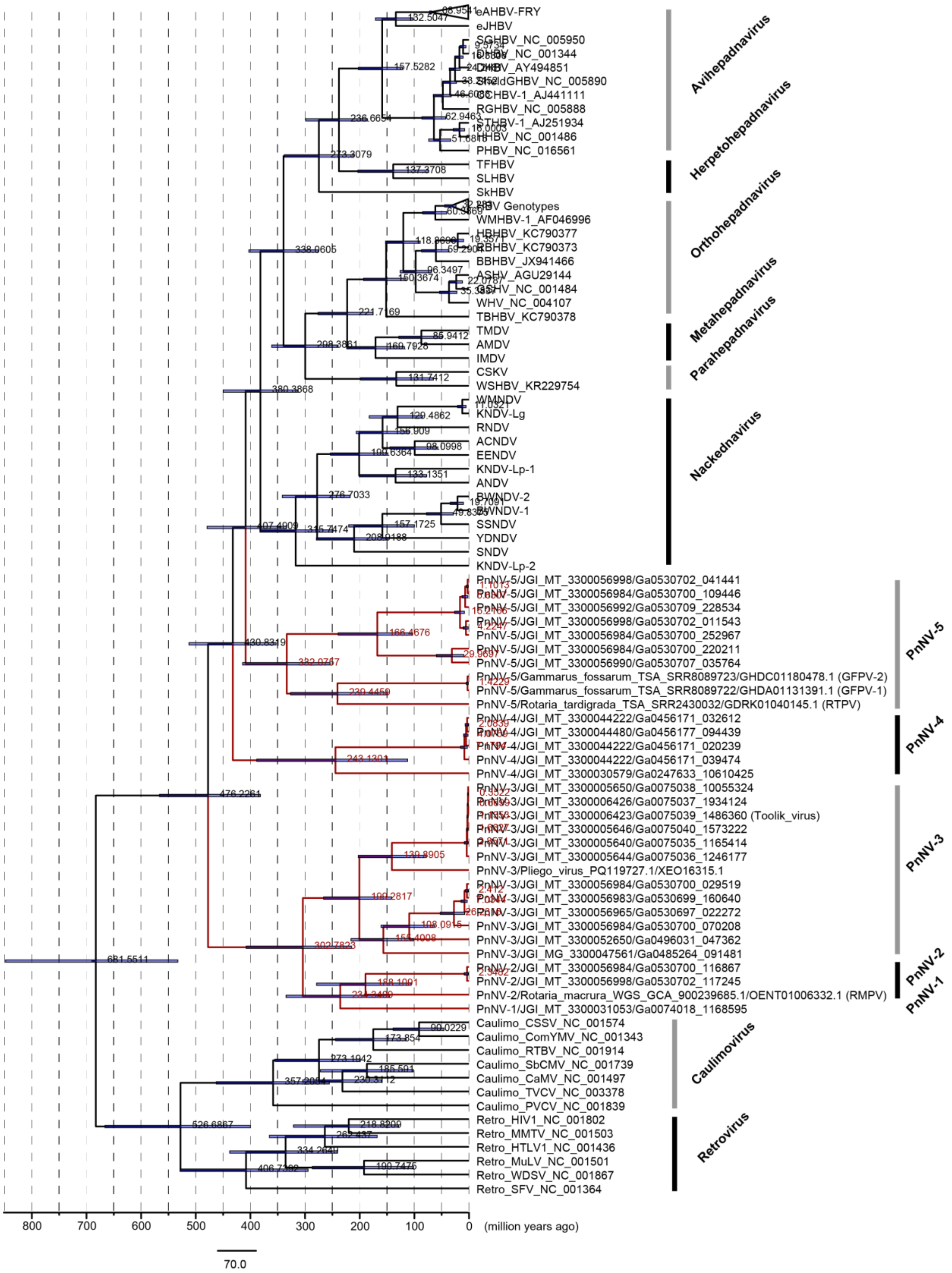

eAHBV-FRY
eJHBV
SGHBV_NC_005950
RGHBV_NC_005888
STHBV-1_AJ251934
PHBV_NC_016561
TFHBV
SLHBV
SkHBV
WMHBV-1_AF046996
BBHBV_JX941466
WHV_NC_004107
TBHBV_KC790378
TMDV
AMDV
IMDV
CSKV
WSHBV_KR229754
KNDV-Lg
RNDV
ACNDV
EENDV
KNDV-Lp-1
ANDV
SSNDV
YDNDV
SNDV
KNDV-Lp-2
PnNV-5/JGI_MT_3300056984/Ga0530700_252967
PnNV-5/JGI_MT_3300056990/Ga0530707_035764
PnNV-5/Gammarus_fossarum_TSA_SRR8089722/GHDA01131391.1 (GFPV-1)
PnNV-5/Rotaria_tardigrada_TSA_SRR2430032/GDRK01040145.1 (RTPV)
PnNV-4/JGI_MT_3300044222/Ga0456171_039474
PnNV-4/JGI_MT_3300030579/Ga0247633_10610425
PnNV-3/JGI_MT_3300005644/Ga0075036_1246177
PnNV-3/Pliego_virus_PQ119727.1/XEO16315.1
PnNV-3/JGI_MT_3300056984/Ga0530700_070208
PnNV-3/JGI_MT_3300052650/Ga0496031_047362
PnNV-3/JGI_MG_3300047561/Ga0485264_091481
PnNV-2/JGI_MT_3300056998/Ga0530702_117245
PnNV-2/Rotaria_macrura_WGS_GCA_900239685.1/OENT01006332.1 (RMPV)
PnNV-1/JGI_MT_3300031053/Ga0074018_1168595
Caulimo_CSSV_NC_001574
Caulimo_ComYMV_NC_001343
Caulimo_RTBV_NC_001914
Caulimo_SbCMV_NC_001739
Caulimo_CaMV_NC_001497
Caulimo_TVCV_NC_003378
Caulimo_PVCV_NC_001839
Retro_HIV1_NC_001802
Retro_MMTV_NC_001503
Retro_HTLV1_NC_001436
Retro_MuLV_NC_001501
Retro_WDSV_NC_001867
Retro_SFV_NC_001364
Avihepadnavirus
Herpetohepadnavirus
Orthohepadnavirus
Metahepadnavirus
Parahepadnavirus
Nackednavirus
PnNV-5
PnNV-4
PnNV-3
PnNV-2
PnNV-1
Caulimovirus
Retrovirus
800
700
600
500
400
300
200
100
0
(million years ago)
70.0

Supplementary Figure S4. Time-calibrated Bayesian tree of hepadnavirus-related elements based on P proteins. PnNVs are highlighted by red branches. Scale bar, million years ago. eAHBV-FRY elements and HBVs are collapsed. Numbers near nodes indicate the divergence time (million years ago). Node bars represent 95% HPD (highest posterior density) of divergence time.

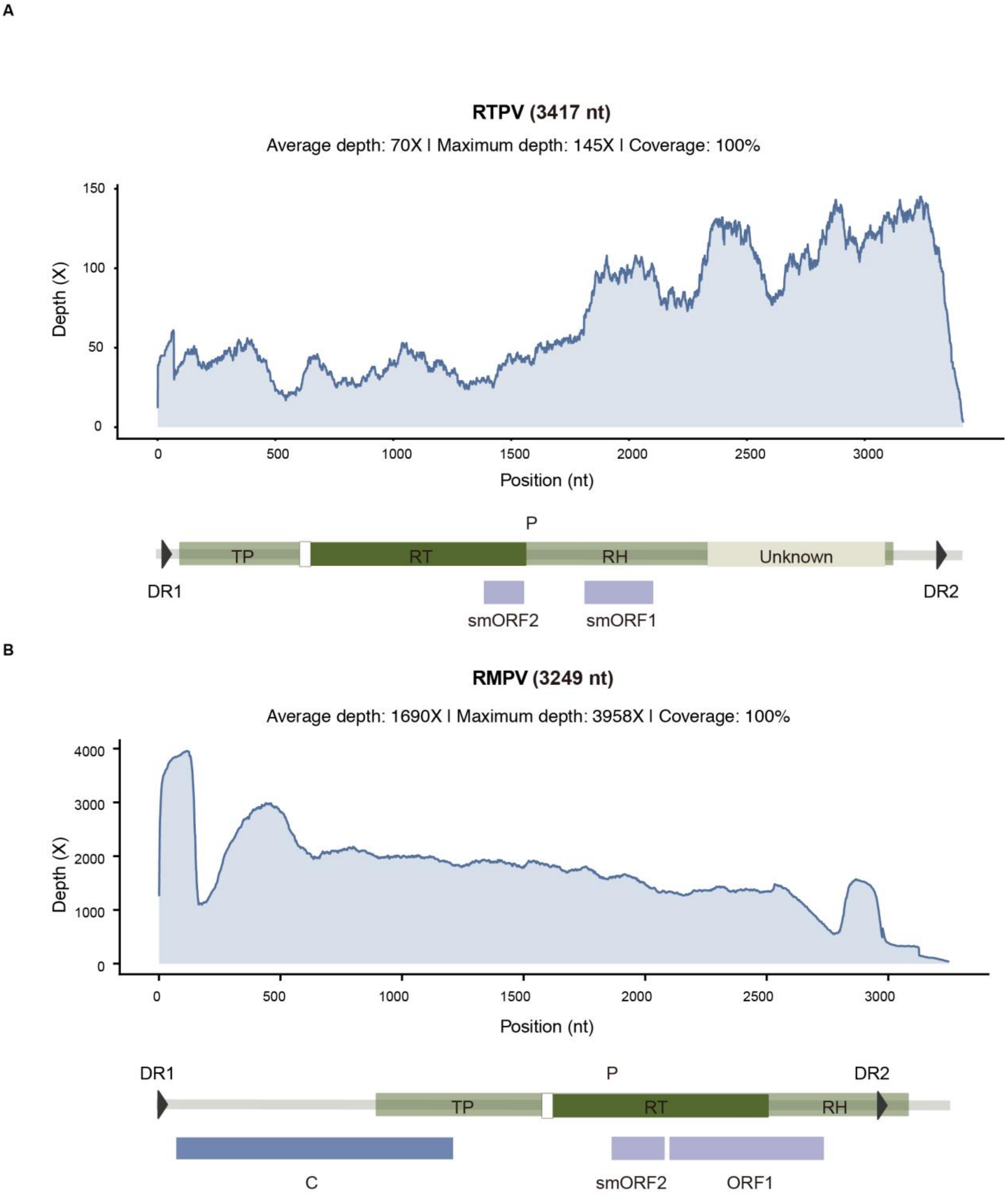

Supplementary Figure S5. Sequencing read coverage along PnNV genomes. Sequencing read depth along the genome of RTPV (A) and RMPV (B). The sequencing depth (top) is aligned with the underlying viral genome architecture (bottom). The y-axis shows the depth of coverage, and the x-axis corresponds to the genomic position.

Supplementary Tables

Supplementary Table S1. Information of PnNVs identified in this study.

Virus Lineage	vOTU Representatives	Genome ID	Length (nt)	Source	Identifier	Assembly Type*	Sequence Type	Sample
PnNV-1	JGI_MT_3300031053/Ga0074018_1168595	JGI_MT_3300031053/Ga0074018_1168595	369	JGI	3300031053	MT	RNA	Forest soil
PnNV-2	JGI_MT_3300056984/Ga0530700_116867	JGI_MT_3300056984/Ga0530700_116867	662	JGI	3300056984	MT	RNA	Peat moss
	JGI_MT_3300056998/Ga0530702_117245	JGI_MT_3300056998/Ga0530702_117245	578	JGI	3300056998	MT	RNA	Peat moss
	Rotaria_macrura_WGS_GCA_900239685.1/OENT01006332.1 (RMPV)	Rotaria_macrura_WGS_GCA_900239685.1/OENT01006332.1	3249	NCBI	GCA_900239685.1	WGS	DNA	*Rotaria macrura*
PnNV-3	JGI_MG_3300047561/Ga0485264_091481	JGI_MG_3300047561/Ga0485264_091481	781	JGI	3300047561	MG	DNA	Freshwater
	JGI_MT_3300052650/Ga0496031_047362	JGI_MT_3300052650/Ga0496031_047362	881	JGI	3300052650	MT	RNA	Peat moss
	JGI_MT_3300006423/Ga0075039_1486360 (Toolik virus)	JGI_MT_3300006423/Ga0075039_1486360	3316	JGI	3300006423	MT	RNA	Permafrost soil
		JGI_MT_3300006426/Ga0075037_1934124	3313	JGI	3300006426	MT	RNA	Permafrost soil
		JGI_MT_3300005646/Ga0075040_1573222	3240	JGI	3300005646	MT	RNA	Permafrost soil
		JGI_MT_3300005650/Ga0075038_10055324	3241	JGI	3300005650	MT	RNA	Permafrost soil
		JGI_MT_3300005640/Ga0075035_1165414	1049	JGI	3300005640	MT	RNA	Permafrost soil

		JGI_MT_3300005644/Ga0075036_1246177	322	JGI	3300005644	MT	RNA	Permafrost soil
	JGI_MT_3300056984/Ga0530700_029519	JGI_MT_3300056984/Ga0530700_029519	1332	JGI	3300056984	MT	RNA	Peat moss
		JGI_MT_3300056983/Ga0530699_160640	454	JGI	3300056983	MT	RNA	Peat moss
	JGI_MT_3300056984/Ga0530700_070208	JGI_MT_3300056984/Ga0530700_070208	856	JGI	3300056984	MT	RNA	Peat moss
	JGI_MT_3300056965/Ga0530697_022272	JGI_MT_3300056965/Ga0530697_022272	1224	JGI	3300056965	MT	RNA	Peat moss
PnNV-4	JGI_MT_3300044480/Ga0456177_094439	JGI_MT_3300044480/Ga0456177_094439	512	JGI	3300044480	MT	RNA	Sediment
	JGI_MT_3300044222/Ga0456171_032612	JGI_MT_3300044222/Ga0456171_032612	736	JGI	3300044222	MT	RNA	Sediment
	JGI_MT_3300044222/Ga0456171_039474	JGI_MT_3300044222/Ga0456171_039474	686	JGI	3300044222	MT	RNA	Sediment
	JGI_MT_3300044222/Ga0456171_020239	JGI_MT_3300044222/Ga0456171_020239	882	JGI	3300044222	MT	RNA	Sediment
	JGI_MT_3300030579/Ga0247633_10610425	JGI_MT_3300030579/Ga0247633_10610425	404	JGI	3300030579	MT	RNA	Forest soil
PnNV-5	JGI_MT_3300056998/Ga0530702_011543	JGI_MT_3300056998/Ga0530702_011543	1884	JGI	3300056998	MT	RNA	Peat moss
		JGI_MT_3300056984/Ga0530700_252967	453	JGI	3300056984	MT	RNA	Peat moss
	JGI_MT_3300056998/Ga0530702_041441	JGI_MT_3300056998/Ga0530702_041441	994	JGI	3300056998	MT	RNA	Peat moss
		JGI_MT_3300056992/Ga0530709_228534	395	JGI	3300056992	MT	RNA	Peat moss

JGI_MT_3300056984/Ga0530700_109446	JGI_MT_3300056984/Ga0530700_109446	684	JGI	3300056984	MT	RNA	Peat moss
JGI_MT_3300056984/Ga0530700_220211	JGI_MT_3300056984/Ga0530700_220211	485	JGI	3300056984	MT	RNA	Peat moss
JGI_MT_3300056990/Ga0530707_035764	JGI_MT_3300056990/Ga0530707_035764	950	JGI	3300056990	MT	RNA	Peat moss
Gammarus_fossarum_TSA_SRR8089722/GHDA01131391.1 (GFPV-1)	Gammarus_fossarum_TSA_SRR8089722/GHDA01131391.1	2223	NCBI	SRR8089722	TSA	RNA	*Gammarus fossarum*
Gammarus_fossarum_TSA_SRR8089723/GHDC01180478.1 (GFPV-2)	Gammarus_fossarum_TSA_SRR8089723/GHDC01180478.1	2102	NCBI	SRR8089723	TSA	RNA	*Gammarus fossarum*
Rotaria_tardigrada_TSA_SRR2430032/GDRK01040145.1 (RTPV)	Rotaria_tardigrada_TSA_SRR2430032/GDRK01040145.1	3417	NCBI	SRR2430032	TSA	RNA	*Rotaria tardigrada*

*WGS: whole genome sequencing; MT: meta-transcriptome; MG: metagenome; TSA: transcriptome shotgun assembly.

Supplementary Table S2. Virus divergence time estimates.

Divergence point	Divergence time (MYA)	
	mean	95% HPD
eAHBV-FRY tMRCA	69	67-71
Avihepadnavirus vs. Herpetohepadnavirus	237	185-298
HBV tMRCA	32	23-43
Orthohepadnavirus vs. Metahepadnavirus	222	174-275
Orthohepadnavirus vs. Parahepadnavirus	298	238-360
Avihepadnavirus vs. Orthohepadnavirus	338	275-402
Hepadnavirus vs. Nackednavirus	380	311-449
Nackednavirus vs. PnNV	407	332-478
Blubervirus tMRCA	476	381-565

Supplementary Datasets

Supplementary Dataset S1. Nucleotide sequences of 32 PnNVs.

Supplementary Dataset S2. Genome annotations of RTPV, RMPV, Toolik virus and Pliego virus, related to Figure 2.

Supplementary Dataset S3. Alignment of core proteins from representative viruses, related to Figure 2.

Supplementary Dataset S4. Alignment of RT domains from representative retroelements, related to Supplementary Figure S1.

Supplementary Dataset S5. Tree file of RT domains from representative retroelements, related to Supplementary Figure S1.

Supplementary Dataset S6. Alignment of RT domains from hepadnavirus-related elements, related to Figure 1A.

Supplementary Dataset S7. Maximum-likelihood tree file of RT domains from hepadnavirus-related elements, related to Figure 1A.

Supplementary Dataset S8. Bayesian tree file of RT domains from hepadnavirus-related elements, related to Figure 1A.

Supplementary Dataset S9. Alignment and time-calibrated tree file of P proteins from hepadnavirus-related elements, related to Figure 1B.

References

1. Altschul SF, Madden TL, Schäffer AA, Zhang J, Zhang Z, Miller W, Lipman DJ. 1997. Gapped BLAST and PSI-BLAST: a new generation of protein database search programs. *Nucleic Acids Res* **25**:3389-3402.
2. Camacho C, Coulouris G, Avagyan V, Ma N, Papadopoulos J, Bealer K, Madden TL. 2009. BLAST+: architecture and applications. *BMC Bioinformatics* **10**:421.
3. Gong Z, Han GZ. 2024. Kuafuorterviruses, a novel major lineage of reverse-transcribing viruses. *Virus Evol* **10**:veae110.
4. Price MN, Dehal PS, Arkin AP. 2010. FastTree 2—approximately maximum-likelihood trees for large alignments. *PLoS One* **5**:e9490.
5. Fu L, Niu B, Zhu Z, Wu S, Li W. 2012. CD-HIT: accelerated for clustering the next-generation sequencing data. *Bioinformatics* **28**:3150-3152.
6. Roux S, Adriaenssens EM, Dutilh BE, Koonin EV, Kropinski AM, Krupovic M, Kuhn JH, Lavigne R, Brister JR, Varsani A, Amid C, Aziz RK, Bordenstein SR, Bork P, Breitbart M, Cochrane GR, Daly RA, Desnues C, Duhaime MB, Emerson JB, Enault F, Fuhrman JA, Hingamp P, Hugenholtz P, Hurwitz BL, Ivanova NN, Labonté JM, Lee KB, Malmstrom RR, Martinez-Garcia M, Mizrachi IK, Ogata H, Páez-Espino D, Petit MA, Putonti C, Rattei T, Reyes A, Rodriguez-Valera F, Rosario K, Schriml L, Schulz F, Steward GF, Sullivan MB, Sunagawa S, Suttle CA, Temperton B, Tringe SG, Thurber RV, Webster NS, Whiteson KL, Wilhelm SW, Wommack KE, Woyke T, Wrighton KC, Yilmaz P, Yoshida T, Young MJ, Yutin N, Allen LZ, Kyrpides NC, Eloe-Fadrosh EA. 2019. Minimum information about an uncultivated virus genome (MIUViG). *Nat Biotechnol* **37**:29-37.
7. Kearse M, Moir R, Wilson A, Stones-Havas S, Cheung M, Sturrock S, Buxton S, Cooper A, Markowitz S, Duran C, Thierer T, Ashton B, Meintjes P, Drummond A. 2012. Geneious Basic: an integrated and extendable desktop software platform for the organization and analysis of sequence data. *Bioinformatics* **28**:1647-1649.

8. Marchler-Bauer A, Bryant SH. 2004. CD-Search: protein domain annotations on the fly. *Nucleic Acids Res* **32**:W327-W331.

9. Jones P, Binns D, Chang HY, Fraser M, Li W, McAnulla C, McWilliam H, Maslen J, Mitchell A, Nuka G, Pesseat S, Quinn AF, Sangrador-Vegas A, Scheremetjew M, Yong SY, Lopez R, Hunter S. 2014. InterProScan 5: genome-scale protein function classification. *Bioinformatics* **30**:1236-1240.

10. Kelley LA, Sternberg MJ. 2009. Protein structure prediction on the Web: a case study using the Phyre server. *Nat Protoc* **4**:363-371.

11. Zimmermann L, Stephens A, Nam SZ, Rau D, Kübler J, Lozajic M, Gabler F, Söding J, Lupas AN, Alva V. 2018. A completely reimplemented MPI bioinformatics toolkit with a new HHpred server at its core. *J Mol Biol* **430**:2237-2243.

12. Holm L. 2022. Dali server: structural unification of protein families. *Nucleic Acids Res* **50**:W210-W215.

13. Jumper J, Evans R, Pritzel A, Green T, Figurnov M, Ronneberger O, Tunyasuvunakool K, Bates R, Žídek A, Potapenko A, Bridgland A, Meyer C, Kohl SAA, Ballard AJ, Cowie A, Romera-Paredes B, Nikolov S, Jain R, Adler J, Back T, Petersen S, Reiman D, Clancy E, Zielinski M, Steinegger M, Pacholska M, Berghammer T, Bodenstein S, Silver D, Vinyals O, Senior AW, Kavukcuoglu K, Kohli P, Hassabis D. 2021. Highly accurate protein structure prediction with AlphaFold. *Nature* **596**:583-589.

14. van Kempen M, Kim SS, Tumescheit C, Mirdita M, Lee J, Gilchrist CLM, Söding J, Steinegger M. 2024. Fast and accurate protein structure search with Foldseek. *Nat Biotechnol* **42**:243-246.

15. Parisien M, Major F. 2008. The MC-Fold and MC-Sym pipeline infers RNA structure from sequence data. *Nature* **452**:51-55.

16. Drozdetskiy A, Cole C, Procter J, Barton GJ. 2015. JPred4: a protein secondary structure prediction server. *Nucleic Acids Res* **43**:W389-W394.

17. Katoh K, Standley DM. 2013. MAFFT multiple sequence alignment software version 7: improvements in performance and usability. *Mol Biol Evol* **30**:772-780.

18. Nguyen LT, Schmidt HA, von Haeseler A, Minh BQ. 2015. IQ-TREE: a fast and effective stochastic algorithm for estimating maximum-likelihood phylogenies. *Mol Biol Evol* **32**:268-274.
19. Kalyaanamoorthy S, Minh BQ, Wong TKF, von Haeseler A, Jermiin LS. 2017. ModelFinder: fast model selection for accurate phylogenetic estimates. *Nat Methods* **14**:587-589.
20. Hoang DT, Chernomor O, von Haeseler A, Minh BQ, Vinh LS. 2018. UFBoot2: improving the ultrafast bootstrap approximation. *Mol Biol Evol* **35**:518-522.
21. Ronquist F, Teslenko M, van der Mark P, Ayres DL, Darling A, Höhna S, Larget B, Liu L, Suchard MA, Huelsenbeck JP. 2012. MrBayes 3.2: efficient Bayesian phylogenetic inference and model choice across a large model space. *Syst Biol* **61**:539-542.
22. Lauber C, Seitz S, Mattei S, Suh A, Beck J, Herstein J, Börold J, Salzburger W, Kaderali L, Briggs JAG, Bartenschlager R. 2017. Deciphering the origin and evolution of Hepatitis B Viruses by means of a family of non-enveloped fish viruses. *Cell Host Microbe* **22**:387-399.e6.
23. Jarvis ED, Mirarab S, Aberer AJ, Li B, Houde P, Li C, Ho SY, Faircloth BC, Nabholz B, Howard JT, Suh A, Weber CC, da Fonseca RR, Li J, Zhang F, Li H, Zhou L, Narula N, Liu L, Ganapathy G, Boussau B, Bayzid MS, Zavidovych V, Subramanian S, Gabaldón T, Capella-Gutiérrez S, Huerta-Cepas J, Rekepalli B, Munch K, Schierup M, Lindow B, Warren WC, Ray D, Green RE, Bruford MW, Zhan X, Dixon A, Li S, Li N, Huang Y, Derryberry EP, Bertelsen MF, Sheldon FH, Brumfield RT, Mello CV, Lovell PV, Wirthlin M, Schneider MP, Prosdocimi F, Samaniego JA, Vargas Velazquez AM, Alfaro-Núñez A, Campos PF, Petersen B, Sicheritz-Ponten T, Pas A, Bailey T, Scofield P, Bunce M, Lambert DM, Zhou Q, Perelman P, Driskell AC, Shapiro B, Xiong Z, Zeng Y, Liu S, Li Z, Liu B, Wu K, Xiao J, Yinqi X, Zheng Q, Zhang Y, Yang H, Wang J, Smeds L, Rheindt FE, Braun M, Fjeldsa J, Orlando L, Barker FK, Jønsson KA, Johnson W, Koepfli KP, O'Brien S, Haussler D, Ryder OA, Rahbek C, Willerslev E, Graves GR, Glenn TC, McCormack J, Burt D, Ellegren H, Alström P, Edwards SV, Stamatakis A, Mindell DP, Cracraft J, Braun EL, Warnow T, Jun W, Gilbert MT, Zhang

G. 2014. Whole-genome analyses resolve early branches in the tree of life of modern birds. *Science* **346**:1320-1331.

24. Darriba D, Taboada GL, Doallo R, Posada D. 2011. ProtTest 3: fast selection of best-fit models of protein evolution. *Bioinformatics* **27**:1164-1165.
25. Suchard MA, Lemey P, Baele G, Ayres DL, Drummond AJ, Rambaut A. 2018. Bayesian phylogenetic and phylodynamic data integration using BEAST 1.10. *Virus Evol* **4**:vey016.
26. Rambaut A, Drummond AJ, Xie D, Baele G, Suchard MA. 2018. Posterior summarization in Bayesian phylogenetics using Tracer 1.7. *Syst Biol* **67**:901-904.
27. Bolger AM, Lohse M, Usadel B. 2014. Trimmomatic: a flexible trimmer for Illumina sequence data. *Bioinformatics* **30**:2114-2120.
28. Langmead B, Salzberg SL. 2012. Fast gapped-read alignment with Bowtie 2. *Nat Methods* **9**:357-359.
